# Editorial: Innovative bioconversion of non-food substrates to fuels

**DOI:** 10.3389/fbioe.2023.1163513

**Published:** 2023-03-03

**Authors:** Thaddeus Chukwuemeka Ezeji, Hasan Atiyeh, Adriano Pinto Mariano, Sudip Kumar Rakshit

**Affiliations:** ^1^ Department of Animal Sciences, The Ohio State University, Columbus, OH, United States; ^2^ Department of Biosystems and Agricultural Engineering, College of Agricultural Sciences and Natural Resources, Oklahoma State University, Stillwater, OK, United States; ^3^ Laboratory of Optimization, Design, and Advanced Control—Fermentation Division (LOPCA-Ferm), School of Chemical Engineering, University of Campinas, (UNICAMP), Campinas, Brazil; ^4^ Department of Chemical Engineering, The Biorefining Research Institute at Lakehead University, Thunder Bay, ON, Canada

**Keywords:** lignocellulosic biomass, butanol, ethanol, syngas, carbondioxide, phenylethanol, butanetriol

Due to the finite quantity of fossil-derived fuels that can be used for energy and chemical production, coupled with the need to reduce greenhouse gas (GHG) emissions, the development and production of biobased fuels and chemicals have attracted the attention of scientists, engineers, and policymakers as a potential strategy for simultaneous maintenance of energy security and the mitigation of GHG emissions in the environment. Bio-derived fuels and chemicals include, but are not limited to, ethanol, butanol, propanol, butanediol, propanediol, lactic acid, succinic acid, acetic acid, butyric acid, hydrogen (H_2_), and methane (CH_4_). Food substrates such as corn and sugarcane are two major renewable substrates that are currently used in large quantities to produce biobased fuels and chemicals. These food substrates also serve as feed for livestock and as consumer products, which creates competition and increases food costs. More economical choices are non-food substrates such as lignocellulosic biomass (LB), inedible food wastes, and gases that are generally considered waste, which are currently being considered as potential alternative feedstocks to produce renewable fuels and chemicals.

The LB is the most abundant renewable resource on the planet, with a global annual production of 181.5 billion tons (Dahmen et al., 2019) and has great potential as a substrate for fermentation because it is composed of more than 75% fermentable polymeric sugars such as glucose, cellobiose, xylose, arabinose, and mannose. LB includes, but is not limited to, corn stover, wheat straw, rice straw and hulls, sugarcane bagasse, Napier grass, giant reed grass, sweet sorghum, willow, switchgrass, *Miscanthus*, eucalyptus, Eastern red cedar, sawdust, wood shavings, and forestry residues, and is a significant component of municipal solid waste (MSW). LB, however, must undergo pretreatment and hydrolysis prior to use in the fermentation of fuels and chemicals. Undesirable lignocellulose-derived microbial inhibitory compounds (LDMICs) that inhibit the growth of fermentation microorganisms are generated during the pretreatment and hydrolysis of LB. LDMICs impede the bioconversion of LB hydrolysates (sugars) to fuels and chemicals and must be either removed prior to use as a fermentation feedstock, fermentation medium modified, fermentation microorganism metabolically engineered, or combinations thereof to increase tolerance to the LDMICs and facilitate the fermentation of LB hydrolysates into fuels and chemicals.

In the supply chain of food produced in the United States, between 30% and 40% of the food produced annually for inclusion in human diets is not used as a source of nutrition (www.usda.gov/foodwaste/faqs). Additionally, about 14% of MSW is solid food waste, which generates annual GHG emissions of at least 113 million metric tons of carbon dioxide (CO_2_) (Venkat, 2012; Ujor et al., 2014). Consequently, numerous researchers are evaluating the best practices to reduce food loss and waste at different points in the supply chain. Global energy-related CO_2_ emissions, however, increased in 2018 to 33.243 gigatons after the cessation of the increased emission trajectory during the 2014–2016 period due to the inability of cleaner energy sources to meet the growing energy demand (https://www.iea.org/reports/world-energy-outlook-2019). In the European Union and many other countries, including the United States, policies are being formulated or initiatives are being proposed to achieve “net-zero” CO_2_ emissions by 2070. While it is conceivable that there will be sustainability in CO_2_ extraction from the atmosphere before 2070, there are doubts about whether this timeline is a realistic goal because many of the technologies for CO_2_ capture and conversion to value-added products such as fuels and chemicals have not been proven to be feasible at pilot and commercial scales (https://www.iea.org/reports/world-energy-outlook-2019).

Theoretically, efficient conversion of LB, food waste, and CO_2_ to fuels and chemicals is within the realm of possibility because in aquatic and terrestrial ecosystems there is an abundance of diverse microorganisms with the capacity of catalyzing an unlimited number of chemical reactions, and the fields of synthetic biology, process engineering, and metabolic engineering have evolved to the point that microorganisms can be induced or manipulated to catalyze foreign or enormously improve indigenous biosynthetic reactions, especially with compatible process technologies (Ezeji, 2017). Regardless of the substrate used, fermentative production of fuels and chemicals has several limitations, such as small titers, low productivities, and uneconomical production quantities due to inadequate technological advancements. Additionally, the fermentative production of fuels and chemicals is mostly accompanied by the production of undesirable CO_2_, a GHG, which contributes to compromised target product yields. Consequently, this Research Topic highlights the understanding and development of knowledge-based strategies to resolve interrelated issues regarding non-food substrates, technological compatibility, and mitigation of greenhouse gas generation.

The six articles in this Research Topic, five of which are research manuscripts and one of which is a review, are focused on themes of the development of process technologies for converting LB, CO_2_ + CO, or CO_2_ to environmentally compatible products such as D-1,2,4-butanediol, 2-phenylethanol (2-PE), acetone, ethanol, butanol, hexanol, and 2,3-butanediol. In one review article, ideas, techniques, and potential pitfalls of CRISPR-based genome editing for modifying *Saccharomyces cerevisiae* to be more useful in producing economically viable and environmentally compatible valuable products through fermentation processes are elaborated.

Consistent with the theme of bioconversion of non-food substrates to fuels and chemicals, the research (Olorunsogbon et al.) evaluated the capacity of genetically engineered *C. beijerinckii* NCIMB 8052 to tolerate the deleterious effects of LDMICs and produce acetone and butanol with hydrothermolysis-pretreated switchgrass hydrolysates. These researchers discovered that metabolic engineering of *C. beijerinckii* enhanced tolerance of LDMICs and, in combination with modifications to the fermentation milieu, increased the production of fuels and chemicals from LB hydrolysates. In addition, Wang et al. contributed an article highlighting the feasibility of producing D-1,2,4-butanediol (BT), a straight-chain non-natural four-carbon polyol that can be used as a propellant and an energetic plasticizer, using LB-derived arabinose and *Escherichia coli*. It is noteworthy that this article is the first report on an alternative biosynthetic pathway to produce BT.

To be able to convert LB, including its lignin component, into value-added products without having to conduct fractionation and saccharification processes is of great interest and would be a great benefit. Gasification of LB to syngas (CO, CO_2_, and H_2_) followed by syngas fermentation is one such approach with the capacity to extract and convert 100% of all carbons in LB into target products. Recognizing the importance of developing cost-effective technologies to convert LB to fuels and chemicals, the members of the team led by Youngsoon Um contributed an article in which process development that focused on modifications of the syngas fermentation milieu resulted in the increase of hexanol production from the current < 1 g/L to 2.34 g/L Oh et al. By using gas analysis, metabolomics, transcriptomics, and metabolic modeling, de Lima et al. evaluated a systems approach to the metabolic response of *C. autoethanogenum* during growth in a CO or syngas (CO + CO_2_ + H_2_) milieu in a chemostat. While the findings from this study could lead to an improved understanding of transcriptional regulation in acetogens, there is a highlighting of the need to map genotype-phenotype associations and improve gene annotations in *C. autoethanogenum* and the need for advancing understanding of acetogen metabolism (de Lima et al.). Similarly, the team led by Alessandro Cordara contributed an article that details the effects of combining metabolic engineering and supplementing growth medium with L-phenylalanine on CO_2_ uptake and conversion to 2-phenylethanol (2-PE) by cyanobacteria. The combination of the two approaches led to a 2-PE yield of 300 mg/gDW and a maximum 2-PE titer of 285 mg/L, a 2.4-fold greater value than that previously reported (Usai et al.); thus, there is a highlighting of the importance of medium composition in the bio-catalysis of substrates to fuels and chemicals.

In the review article in this Research Topic, there is an overview of inconspicuous but important details that need to be considered when attempting to improve the efficiency of CRISPR/Cas9 genome editing technologies in *S. cerevisiae* to enhance the production of fuels, chemicals, and proteins (Antony et al.). In this article, there is a focus on the important considerations and mechanics of various CRISPR principles and technologies, such as pre-CRISPR marker-free genome editing, marker-free genome editing with CRISPR/Cas9, cellular factors affecting Cas9 genome editing outcomes, multiplex genome editing with CRISPR, practical considerations for CRISPR genome editing, allele-specific genome editing in diploid yeast, alternative CRISPR-based applications for manipulating the genome of *S. cerevisiae*, and strategies to reduce off-target binding and cleavage by Cas proteins in *S. cerevisiae*.

Taken as a whole, we anticipate that this Research Topic will serve as a valuable resource on a global scale for agricultural, chemical, and bioprocess engineers, systems analysts, microbiologists, students, technicians, farmers, biorefineries, government agencies, researchers, and postdoctoral research associates with focused interests and commitments to developing new effective and efficient technologies for the conversion of non-food substrates to value-added products such as fuels and feedstock chemicals.
